# Nitrofurantoin-Induced Pulmonary Toxicity: Mechanisms, Diagnosis, and Management

**DOI:** 10.3390/toxics13050382

**Published:** 2025-05-09

**Authors:** Alan D. Kaye, Shivam S. Shah, Leon LaHaye, John A. Hennagin, Anna K. Ardoin, Alexandra Dubuisson, Shahab Ahmadzadeh, Sahar Shekoohi

**Affiliations:** 1 Departments of Anesthesiology and Pharmacology, Toxicology and Neurosciences, Louisiana State University Health Sciences Center Shreveport, Shreveport, LA 71103, USA; 2 School of Medicine, Louisiana State University Health Sciences Center at Shreveport, Shreveport, LA 71103, USA; sss002@lsuhs.edu (S.S.S.); jah002@lsuhs.edu (J.A.H.); adu001@lsuhs.edu (A.D.); 3 Department of Anesthesiology, Louisiana State University Health Sciences Center Shreveport, Shreveport, LA 71103, USA

**Keywords:** nitrofurantoin, pulmonary toxicity, interstitial lung disease, drug-induced hypersensitivity, adverse reaction

## Abstract

Nitrofurantoin, a commonly prescribed antibiotic for urinary tract infections, has been associated with rare but potentially serious pulmonary toxicity, which can present in acute, subacute, or chronic forms. Acute toxicity typically manifests in the form of hypersensitivity pneumonitis, which is characterized by fever, dyspnea, and eosinophilia, often resolving rapidly after drug discontinuation. However, chronic toxicity can lead to interstitial lung disease with progressive fibrosis, causing significant and sometimes irreversible pulmonary impairment. The pathophysiology of nitrofurantoin-induced lung injury is thought to involve oxidative stress, immune-mediated mechanisms, and direct cytotoxic effects; however, the exact pathways remain incompletely understood. Clinical diagnosis is challenging due to nonspecific symptoms that often resemble other respiratory conditions, leading to delays in recognition and treatment. Radiographic findings vary, with acute cases showing diffuse ground-glass opacities, while chronic cases may demonstrate reticular interstitial changes and fibrosis. The discontinuation of nitrofurantoin is the primary intervention, but corticosteroids may be beneficial, particularly in chronic cases with persistent inflammation or fibrosis, though their efficacy remains uncertain. Given the risk of long-term respiratory complications, heightened awareness among healthcare providers is essential for early diagnosis and intervention. Future research is needed to better define risk factors, improve diagnostic criteria, and explore alternative treatment strategies that mitigate the potential for pulmonary toxicity while maintaining effective antimicrobial therapy. This review explores the pathophysiology, clinical presentation, diagnostic challenges, and management strategies for nitrofurantoin-induced pulmonary toxicity.

## 1. Introduction

Nitrofurantoin, a broad-spectrum antibiotic, has been a key component of the treatment of uncomplicated urinary tract infections (UTIs), particularly in women [1]. First introduced in the 1950s, it is commonly used for its efficacy, low resistance profile, and targeted action against uropathogenic bacteria. Although its mechanism is still unclear, the drug may work by blocking bacterial protein synthesis by targeting ribosomal proteins through reactive intermediates, as well as suppressing inducible enzyme synthesis [2]. Its multiple mechanisms make it difficult for bacteria to develop resistance. Due to its established utility and favorable safety profile in the majority of patients, nitrofurantoin continues to be widely prescribed for UTIs. However, like all medications, it has potential adverse effects. While pulmonary reactions to nitrofurantoin are rare, they are the most commonly reported chronic side effects, with long-term use increasing the likelihood of these reactions compared to other adverse effects, such as liver or nerve toxicity [3].

Pulmonary toxicity associated with nitrofurantoin, though rare, is a well-documented and potentially serious complication. The incidence of acute pulmonary toxicity is approximately 1 in 5000 patients after initial exposure, with severe respiratory impairment requiring hospitalization occurring in about 1 in 750 long-term users [4]. The vast majority of affected individuals (85–94%) are women, due to their higher rates of recurrent urinary tract infections and, subsequently, their greater exposure to nitrofurantoin [5,6]. Due to its renewed use as a first-line urinary antiseptic, the prevalence of nitrofurantoin-induced pulmonary injury has seen a resurgence. This adverse effect can manifest as acute or chronic pulmonary reactions, ranging from mild symptoms like cough and dyspnea to more severe outcomes such as interstitial lung disease, pulmonary fibrosis, and even acute respiratory distress syndrome [7]. Nitrofurantoin-induced pulmonary toxicity is believed to result from a combination of immune-mediated and direct toxic effects on the lung tissue, potentially exacerbated by predisposing factors such as patient genetics [8].

Recognizing and managing nitrofurantoin-induced pulmonary toxicity is essential due to the potential for rapid progression to severe, irreversible lung damage. The early identification of symptoms, such as unexplained cough, chest pain, or shortness of breath, is needed to promptly discontinue the drug and initiate appropriate treatment. In some cases, immediate cessation may lead to complete resolution, while in others, particularly those with chronic or severe reactions, management may require other interventions [9].

Therefore, the present investigation explores the mechanisms, clinical manifestations, and management strategies of nitrofurantoin-induced pulmonary toxicity, with a focus on early diagnosis, prevention, and therapeutic approaches to reduce long-term complications. Understanding these risks is crucial for physicians to ensure both the safe and effective use of nitrofurantoin in clinical practice.

## 2. Mechanism of Toxicity

### 2.1. Nitrofurantoin Metabolism and Pathways Involved in Lung Tissue Damage

**Figure 1:**
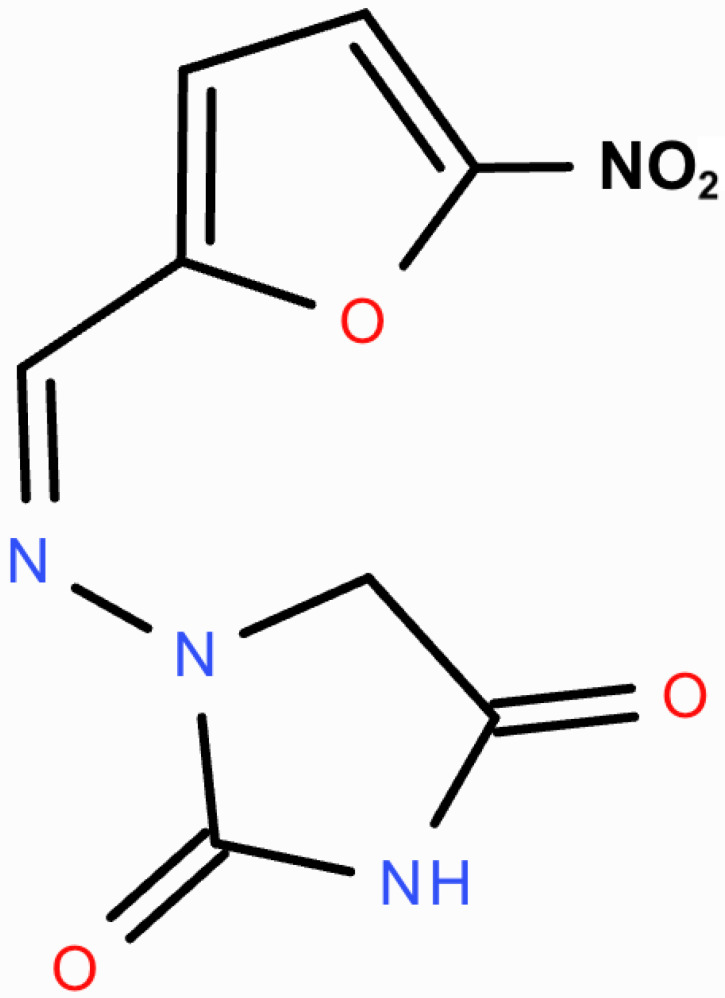
Chemical structure of nitrofurantoin.

**Figure 2:**
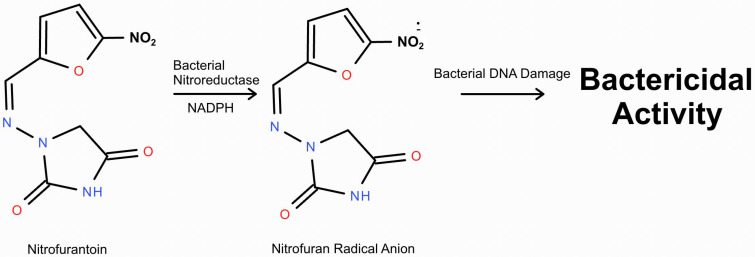
Mechanism of nitrofurantoin’s bactericidal properties.

Nitrofurantoin, a bacterial antibiotic primarily used for urinary tract infections, undergoes metabolism predominantly in the liver through reduction and oxidation pathways (Figure 1). It is a prodrug that requires nitroreductase activation, which then reduces its nitro group into reactive intermediates (Figure 2) [10]. These oxidative intermediates interact with bacterial DNA, leading to the disruption of essential cellular functions of the bacteria [10]. The primary metabolic byproducts of nitrofurantoin include reactive oxygen species (ROS) and hydroxylamine derivatives, which are integral to its bactericidal activity [10]. However, the production of ROS is not limited to bacterial cells; in human tissues, excessive ROS generation can lead to oxidative stress, especially in oxygen-rich environments such as the lungs [10]. Factors like impaired drug clearance or increased drug concentrations can heighten systemic ROS levels, making metabolism-related side effects more likely.

Nitrofurantoin-induced lung toxicity, both acute and chronic, is mediated through oxidative stress and immune activation pathways. The ROS generated during metabolism causes lipid peroxidation, protein denaturation, and DNA damage, contributing to cellular injury in the lung epithelium [10]. This oxidative damage triggers the activation of inflammatory pathways, including cytokine release and leukocyte infiltration [11]. Chronic exposure or hypersensitivity to nitrofurantoin metabolites can result in interstitial lung disease, which is related to fibroblast proliferation and extracellular matrix deposition. Additionally, immune-mediated hypersensitivity responses, characterized by increased levels of eosinophils and immunoglobulins, exacerbate lung damage in susceptible individuals [11]. Understanding these pathways is crucial for managing adverse reactions and tailoring therapeutic approaches to minimize the risk of pulmonary toxicity associated with nitrofurantoin.

### 2.2. Potential Immunologic and Oxidative Stress Mechanisms

Nitrofurantoin-induced pulmonary toxicity is mostly understood by involving oxidative stress and immune-mediated injury. The metabolism of nitrofurantoin generates superoxide anion free radicals, which lead to oxidative stress in lung tissue [10]. This oxidative damage disrupts the structural integrity of cellular membranes via lipid peroxidation and induces protein and DNA modifications, triggering cell apoptosis or necrosis [10]. An experimental study in rats found that nitrofurantoin-induced pulmonary toxicity is driven by oxidative stress, due to its increased lipid peroxidation and decreased GSH/GSSG ratios [12]. The accumulation of reactive oxygen species, including hydrogen peroxide, overwhelms the lung’s antioxidant defenses, leading to membrane damage and cell dysfunction. The reduced activity of antioxidants like glutathione reductase and catalase further exacerbates the oxidative damage. This results in endothelial dysfunction, as shown by decreased ACE activity, and contributes to pulmonary edema. The toxicity is heightened in rats with deficiencies in antioxidants, such as vitamin E and glutathione, suggesting that compromised antioxidant systems amplify nitrofurantoin-induced lung injury [12].

Additionally, nitrofurantoin metabolites may function as haptens, binding to host proteins and eliciting an adaptive immune response [13]. It has also been found that patients with nitrofurantoin toxicity have increased levels of eosinophils and serum immunoglobin E, further indicating an inflammatory response and hypersensitivity reactions, which intensifies tissue injury [11]. This typically leads to an acute pulmonary reaction. However, the exact mechanism is still unknown. Possible proposed mechanisms for this reaction include the idea that it may be an immune-complex-mediated response, a cytotoxic response, or a cell-mediated reaction [11]. Chronic exposure to these mechanisms can contribute to the development of interstitial lung fibrosis. The resulting pulmonary fibrosis may be a result of the recruitment of fibroblasts by the immune system; however, in a case report evaluating nitrofurantoin-induced pulmonary fibrosis, fibroblast foci were not seen [14]. Nitrofurantoin can alter the behavior of lung fibroblasts, inducing a phenotype that is primed for extracellular matrix (ECM) repair. In a study by Leslie et al., treatment with nitrofurantoin increased fibroblast motility and significantly changed cell morphology by enlarging the cell area and decreasing its roundness, which are features that are characteristic of activated or proto-myofibroblastic cells [15]. This study also found increased fibronectin deposition at higher concentrations of the drug (20 μg/mL), which is a protein that is known to support ECM organization and promote pro-fibrotic signaling. These changes suggest that nitrofurantoin may enhance fibroblast-mediated ECM deposition and remodeling, potentially leading to increased tissue stiffness and fibrotic progression over time. It is believed that the oxidative damage from nitrofurantoin toxicity may lead to lymphocytes producing mediators that lead to the release of cytokines, resulting in lymphocytic alveolitis [14]. However, this mechanism is still unknown, but may be due to a significant increase in the absolute number and percentage of T-helper lymphocytes rather than an increase in T-suppressor lymphocytes [16].

### 2.3. Predisposing Factors

Predisposing risk factors for acute/subacute nitrofurantoin toxicity can include pre-existing respiratory conditions such as asthma or chronic obstructive pulmonary disease (COPD). The significantly acute reactions to nitrofurantoin (within a few hours) are believed to be caused by hypersensitivity reactions [17]. It was found that acute reactions to nitrofurantoin occur in around 1 in 5000 cases after first exposure, suggesting that the acute response is very rare [11]. For the subacute reactions (days to weeks), it is believed that respiratory conditions may heighten susceptibility by impairing the ability of the lung to respond to and to recover from oxidative and inflammatory stress [11]. Another predisposing condition is glucose-6-phosphate dehydrogenase deficiency (G6PD) [18]. Patients who have G6PD already have an increase in the number of free radicals present within cells; the use of nitrofurantoin exacerbates this issue, leading to hemolytic anemia and possibly pulmonary toxicity, where even more free radicals are present [18].

Chronic nitrofurantoin pulmonary toxicity is most commonly seen in older women. This is because the risk of UTIs is most prevalent in women with a median age of 60 to 70 years old [19]. A retrospective review by Santos et al. found that age plays a significant role in the risk of lung injury from nitrofurantoin, with individuals aged 75–84 years and those aged ≥ 85 years having a significantly higher risk compared to those aged 65–74 years [20]. Additionally, this study found that male sex is not significantly associated with an increased risk of lung injury when compared to female sex [20]. Nitrofurantoin is prescribed as a prophylactic to avoid UTIs, and after months to years of use, the symptoms of nitrofurantoin pulmonary toxicity can occur. One of the major risk factors for nitrofurantoin toxicity is the duration of use, because chronic exposure to nitrofurantoin has a 53% greater risk of lung injury compared to acute usage [20]. Typical symptoms of chronic nitrofurantoin toxicity include tachypnea, fever, nonproductive cough, tachycardia, chest pain, rash, and arthralgia [11]. These symptoms are likely the result of oxidative damage that has occurred due to the increase in ROS produced from nitrofurantoin metabolism [11]. The prevalence of developing toxicity in older women is also believed to be due to the decrease in the ability for renal clearance of the drug. However, an observational study that evaluated 38 patients with varying ages and renal functions found no statistically significant difference between varying levels of exposure in the urine [21]. This is likely due to the study’s small size and inability to evaluate elderly women with an estimated glomerular filtration rate (eGFR) < 50 mL/min because of physicians’ reluctance to prescribe nitrofurantoin to these patients [21]. As with any drug that is excreted through the urine, the maintenance dose can be significant to avoid toxicity. When evaluating the maintenance dose, it is important to consider other drug interactions, such as drugs that could acidify the urine, leading to decreased excretion.

### 2.4. Types of Nitrofurantoin-Induced Pulmonary Toxicity

Both acute and chronic nitrofurantoin-induced pulmonary toxicity (NIPT) have been reported [11,19,22]. Though these reactions are rare, they can be life-threatening if not promptly diagnosed and treated [11]. Women are more frequently affected, likely due to the higher prevalence of UTIs in this population.

The acute form of NIPT typically presents within hours to weeks of nitrofurantoin administration with symptoms including fever, cough, dyspnea, and interstitial pneumonitis, often followed by eosinophilia or skin manifestations [23]. The incidence of acute NIPT is estimated at approximately 1 in 5000 cases [24]. This form is primarily associated with an Arthus Type III hypersensitivity reaction in the lungs, although evidence suggests other mechanisms, such as Type II cytotoxicity, Type IV cell-mediated immunity, and direct toxic injury through the formation of oxygen free radicals [24]. These immune responses appear to be independent of dosage [25]. Inspiratory crackles, especially in the lower lung fields, are commonly heard on auscultation [11]. Histopathological examination typically reveals interstitial inflammation, focal hemorrhages, and the presence of eosinophils [26]. Chest X-rays may be normal or show bilateral interstitial infiltrates and pleural effusions, while CT scans often reveal bilateral ground-glass opacities [27,28]. Pulmonary biopsy is rarely required but may show vasculitis, interstitial inflammation, and alveolar exudation [26].

Stopping nitrofurantoin usually results in rapid symptom improvement within 24 h, though eosinophilia may persist for weeks [11]. Chest X-ray findings may take months to resolve fully [24]. If symptoms do not improve after several days, alternative diagnoses should be considered, and further testing may be necessary.

The chronic form of NIPT develops after 6 months or longer of nitrofurantoin use, presenting with progressive dyspnea and cough [29]. These symptoms are generally less severe than in the acute form, and fever is not typically present [26,30]. The chronic form occurs 10–20 times less frequently than the acute form and is more commonly seen in older patients [19,24]. Having the acute form does not predispose patients to the chronic form later [19]. Unlike the acute form, chronic NIPT is thought to result from direct drug toxicity, likely due to the formation of oxygen free radicals, and may be dose dependent.

Histopathological findings may include diffuse interstitial pneumonitis, vascular sclerosis, and chronic interstitial inflammation [26]. Chest X-ray typically shows bilateral interstitial infiltrates, while CT scans may reveal bilateral ground-glass opacities, subpleural irregular linear opacities, honeycombing, traction bronchiectasis, inter- and intralobular septal thickening, and patchy consolidations [6,28,31].

Prompt cessation of nitrofurantoin generally leads to a good prognosis, but diagnosing chronic NIPT can be challenging. Patients are often initially treated for conditions like pulmonary embolism, pneumonia, or heart failure, leading to diagnostic delays that can increase morbidity and mortality [32]. The use of glucocorticoids in treatment remains under study, although many patients recover without them [31,33,34]. It may take weeks to months for symptoms and imaging findings to return to normal after the discontinuation of the drug. The acute and chronic forms of NIPT differ primarily in their timing of onset, clinical severity, and underlying mechanisms (Table 1).

- Timing: Acute NIPT presents within weeks of starting treatment, while chronic NIPT develops after 6 months of sustained, lower-dose use [23,29].
- Severity: Both forms present with dyspnea and cough, but symptoms in the chronic form tend to be less intense, and fever is generally absent in chronic NIPT [23,26,29,30].
- Mechanism: Acute NIPT is mainly due to an Arthus Type III hypersensitivity reaction, whereas chronic NIPT is thought to result from direct toxicity, which is potentially dose-dependent, involving oxygen free radicals [24,26].
- Eosinophilia: This is more common in the acute form, though it can occur in both types [26].
- Imaging: Both forms may show bilateral interstitial infiltrates on chest X-ray [27,28]. CT scans in both forms reveal ground-glass opacities, but the chronic form may also show honeycombing, patchy consolidations, and subpleural irregularities [6,27,28].
- Differential Diagnosis: Conditions such as heart failure, community-acquired pneumonia, and exacerbations of asthma or COPD should be ruled out when considering interstitial lung disease [32].

Management for both forms involves the immediate discontinuation of nitrofurantoin, which leads to rapid improvement in the acute form. However, the chronic form may take months to show improvement, and recovery can be more prolonged [11,24,31,33,34].

## 3. Clinical Presentation and Diagnosis

### 3.1. Symptoms and Clinical Signs Associated with Nitrofurantoin-Induced Pulmonary Toxicity

Nitrofurantoin-induced pulmonary toxicity presents a diagnostic challenge related to its diverse clinical manifestations [9]. This condition can occur as either an acute or chronic response, each with distinct timelines and characteristics [35]. The acute response is believed to be related to a hypersensitivity reaction to the medication, while chronic toxicity is believed to be due to a toxic or cell-mediated reaction [36]. In acute cases, symptoms typically arise within hours to weeks after initiating a short course of nitrofurantoin, whereas chronic reactions may develop months or even years following prolonged use [35]. Both forms of toxicity often present with nonspecific symptoms, such as dry cough, dyspnea, and fever, which overlap with those of other pulmonary disorders [9]. This can lead to misdiagnosis or delayed treatment, complicating the clinical management of affected patients [37].

### 3.2. Diagnostic Approach to Nitrofurantoin-Induced Pulmonary Toxicity

The diagnosis of nitrofurantoin-induced pulmonary toxicity relies on a thorough drug history and the identification of characteristic patterns via pulmonary function testing, histopathological analysis, and chest CT imaging [37]. Pulmonary function tests (PFTs) in affected patients often reveal reduced total lung capacity (TLC), representing the lung volume at maximum inspiration, and a diminished residual volume (RV), whereby the air remains in the lungs after full exhalation. To ensure an accurate diagnosis, PFTs should be performed both before discontinuing nitrofurantoin and three months after its cessation to assess improvement [37].

The acute and chronic forms of nitrofurantoin toxicity exhibit distinct differences in imaging and diagnostic tests despite symptomatic similarities. On chest CT, acute reactions typically present with bilateral ground-glass opacities [34]. In contrast, chronic reactions may show similar findings alongside fibrosis with no defined distribution. Additionally, some cases may exhibit an organizing pneumonia pattern or a mixed pattern of organizing pneumonia and nonspecific interstitial pneumonia [34]. Histopathological features also vary between acute and chronic reactions. Acute cases often reveal mild interstitial inflammation, eosinophilia, focal hemorrhage, and small organizing microthrombi, while chronic cases are characterized by interstitial inflammation, fibrosis, thickened alveolar septa, and vascular sclerosis [37].

### 3.3. Comparison of Nitrofurantoin-Induced Pulmonary Toxicity to Other Pulmonary Conditions

The nonspecific symptoms of nitrofurantoin-induced pulmonary toxicity can mimic those of other pulmonary diseases, such as hypersensitivity pneumonitis (HP) and idiopathic pulmonary fibrosis (IPF) (Table 2). Hypersensitivity pneumonitis (HP), an interstitial lung disease triggered by inhaled exposures, shares imaging and histopathological features with nitrofurantoin-induced pulmonary toxicity [38]. Both conditions can present with ground-glass opacities and occasional fibrosis on chest CT imaging, as well as inflammation and fibrosis on histopathology, particularly in chronic cases [39]. However, key distinguishing features of HP include centrilobular nodules and mosaic attenuation observed on CT scans, which are atypical in nitrofurantoin toxicity. Moreover, diagnostic tools such as serum IgG testing for antigen exposure and biopsy findings of granulomas can support a diagnosis of HP over nitrofurantoin-related injury [38].

Idiopathic pulmonary fibrosis (IPF), a progressive fibrosing interstitial pneumonia with a poor prognosis, can also mimic nitrofurantoin-induced pulmonary injury [40]. While both conditions share symptoms of dry cough and dyspnea, IPF is often also associated with fatigue, nail clubbing, and bibasilar dry inspiratory crackles [40,41]. Imaging findings for IPF may overlap with nitrofurantoin-induced toxicity, but hallmark features such as enlarged mediastinal lymph nodes are characteristic of IPF and are absent in nitrofurantoin-related cases [41].

The response to treatment also provides a key differentiator between nitrofurantoin-induced pulmonary toxicity and other conditions. Acute nitrofurantoin-induced toxicity often resolves rapidly after drug cessation, with symptom improvement observed within 24 h in many cases [37]. Chronic cases also demonstrate significant improvement within three months of stopping the drug, a sharp contrast to the progressive nature of conditions like IPF [34]. This rapid recovery highlights the reversibility of nitrofurantoin-induced toxicity, setting it apart from many other interstitial lung diseases [9].

## 4. Management and Treatment of Nitrofurantoin-Induced Pulmonary Toxicity

Nitrofurantoin-induced pulmonary toxicity, though rare, requires timely and individualized management due to its potential to present acutely as hypersensitivity pneumonitis or chronically as pulmonary fibrosis [42]. The primary intervention is the immediate cessation of nitrofurantoin, where chronic toxicity resolved spontaneously despite severe symptoms. This underscores the potential for recovery even without pharmacological intervention in some cases, though careful monitoring is essential to guide treatment decisions.

The pulmonary adverse effects of nitrofurantoin range significantly in severity, from mild hypersensitivity reactions to severe, irreversible pulmonary fibrosis, which can culminate in respiratory failure and even death [42]. Hypersensitivity reactions may present with transient symptoms such as fever, dyspnea, and cough, often resolving upon the discontinuation of the drug and symptomatic management [43]. For these milder cases, stopping nitrofurantoin and providing supportive care, such as oxygen therapy if needed, typically suffice to achieve symptom resolution [44]. Dosages are tailored based on clinical severity, with short-term therapy often sufficient for acute cases.

Corticosteroids, such as prednisone, are typically employed to reduce inflammation, particularly in acute hypersensitivity reactions [45]. Prednisone is also known to promote the faster resolution of pulmonary lesions [46]. However, while clinical observations suggest that corticosteroids may benefit patients with severe toxicity, strong evidence from controlled clinical trials confirming their routine effectiveness is still needed. This lack of definitive data requires individualized treatment approaches based on the severity of the reaction, patient-specific factors, and the clinical judgment of the treating physician.

In contrast, severe pulmonary toxicity, such as interstitial fibrosis, requires more aggressive intervention [17]. Chronic toxicity poses greater challenges, often requiring pulmonary rehabilitation, which includes supervised exercise programs, breathing techniques, and nutritional guidance to improve overall respiratory function. Additionally, more advanced cases of chronic pulmonary fibrosis may require supplemental oxygen [36,47]. Although antifibrotic therapies, such as pirfenidone and nintedanib, have proven effective in treating non-idiopathic pulmonary fibrosis and other interstitial lung diseases, their efficacy in managing nitrofurantoin-induced pulmonary toxicity remains uncertain [48]. Regular follow-ups are critical in both acute and chronic cases to monitor symptom progression, using pulmonary function tests and imaging studies to identify complications like persistent fibrosis or pulmonary hypertension. Patient education on symptom awareness, such as recognizing new or worsening dyspnea, is essential to ensure timely intervention.

The prognosis varies based on the presentation and management timeline. Acute cases generally have favorable outcomes with early recognition and treatment, while chronic cases may lead to irreversible damage. Nonetheless, ongoing respiratory assessments and tailored management strategies can optimize the quality of life and avoid complications [49]. Spontaneous recovery in some chronic cases highlights the importance of personalized care and close observation [45].

Nitrofurantoin-induced pulmonary toxicity requires a multifaceted approach centered on drug cessation, supportive care, potential corticosteroid use, pulmonary rehabilitation, and regular follow-ups. With timely intervention and patient-centered management, outcomes can be significantly improved, demonstrating the importance of clinician awareness and patient education. Future research is needed to establish standardized guidelines for managing nitrofurantoin-induced pulmonary toxicity, including the role of corticosteroids and other potential interventions.

## 5. Preventive Strategies and Risk Minimization for Nitrofurantoin-Induced Pulmonary Toxicity

Nitrofurantoin is a valuable antibiotic for managing UTIs, but use requires careful strategies to minimize risks (Table 3). High-risk populations, like elderly patients and those with renal impairment, are especially susceptible to adverse effects like pulmonary or hepatic toxicity. In these groups, the drug’s systemic accumulation due to reduced excretion increases the likelihood of harm [50]. Clinicians should assess renal function using eGFR and avoid prescribing nitrofurantoin to patients with eGFR levels below 30 mL/min to avoid these risks.

The long-term use of nitrofurantoin, often required for recurrent UTIs, requires diligent monitoring. Baseline and periodic evaluations of renal, hepatic, and pulmonary function help detect early signs of toxicity. Following prescribing guidelines ensures that nitrofurantoin is used carefully, prioritizing safety, especially in patients needing prolonged treatment. Preventive strategies include utilizing alternative treatments for recurrent UTIs, such as non-antibiotic measures (e.g., probiotics or lifestyle modifications) to mitigate the need for prolonged nitrofurantoin use [51]. Educating healthcare providers about these protocols can enhance these preventive measures.

For recurrent UTI management, alternatives to nitrofurantoin include antibiotics, like trimethoprim-sulfamethoxazole, fosfomycin, or cephalexin, which are selected based on bacterial resistance patterns and individual patient needs [52]. Non-pharmacological options, such as probiotics to promote healthy urinary microbiota, lifestyle modifications like increased hydration, and dietary adjustments, can also play a significant role in reducing recurrence. Adjuncts like cranberry supplements and D-mannose may offer additional preventive benefits, though their effectiveness varies.

Effective decision-making involves weighing nitrofurantoin’s benefits against its risks, particularly in at-risk populations [53]. Shared decision-making empowers patients to understand their options, promoting informed choices about their care. By identifying high-risk individuals, adhering to monitoring protocols, and considering alternative therapies, clinicians can ensure the safe and effective use of nitrofurantoin while reducing potential adverse outcomes.

## 6. Conclusions

Nitrofurantoin-induced pulmonary toxicity remains a rare but clinically significant adverse effect, manifesting in both acute and chronic forms, each presenting distinct diagnostic and management challenges. The underlying mechanisms primarily involve oxidative stress, immune-mediated injury, and direct toxicity from reactive oxygen species generated during nitrofurantoin metabolism. While acute toxicity typically resolves with drug discontinuation, chronic forms develop insidiously over prolonged use, potentially leading to irreversible interstitial fibrosis. Certain patient populations, particularly older women and those with predisposing conditions, may be at increased risk. The nonspecific nature of symptoms and the overlap of imaging findings with other pulmonary disorders often contribute to diagnostic delays. Although the discontinuation of nitrofurantoin is the primary intervention, the role of corticosteroids in chronic cases remains controversial and needs further study. Future research should focus on identifying genetic or molecular markers that predict susceptibility to nitrofurantoin-induced lung injury and exploring safer alternative antibiotics for high-risk patients. Ultimately, optimizing patient safety requires a careful balance between the therapeutic benefits of nitrofurantoin and its potential for severe pulmonary complications, emphasizing individualized risk assessment and vigilant monitoring.

## Figures and Tables

**Table 1:** Key clinical, radiographic, and pathological differences between acute and chronic forms of nitrofurantoin-induced pulmonary toxicity.

Feature	Acute Nitrofurantoin-Induced Pulmonary Toxicity	Chronic Nitrofurantoin-Induced Pulmonary Toxicity
**Onset**	Within hours to weeks of use	After ≥6 months of use
**Symptoms**	Fever, cough, dyspnea, eosinophilia	Progressive cough, dyspnea; usually no fever
**Mechanism**	Immune-mediated (Type III hypersensitivity), ROS-related injury	Direct toxicity via ROS; possibly dose-dependent
**Imaging (CT)**	Ground-glass opacities may be normal	Ground-glass opacities, honeycombing, fibrosis features
**Histopathology**	Interstitial inflammation, eosinophils, vasculitis	Chronic interstitial pneumonitis, vascular sclerosis
**Response to Stopping Drug**	Rapid symptom improvement (24–72 h); slower radiographic resolution	Gradual improvement over weeks to months
**Prognosis**	Excellent with prompt recognition	Variable; often delayed diagnosis
**Treatment**	Stop nitrofurantoin ± steroids	Stop nitrofurantoin ± steroids

**Table 2:** Key comparisons between nitrofurantoin-induced pulmonary toxicity, hypersensitivity pneumonitis, and idiopathic pulmonary fibrosis.

Feature	Nitrofurantoin-Induced Pulmonary Toxicity	Hypersensitivity Pneumonitis	Idiopathic Pulmonary Fibrosis
**Cause**	Nitrofurantoin exposure	Inhaled organic or chemical antigens	Idiopathic
**Symptoms**	Dry cough, dyspnea	Cough, dyspnea, fever, chills	Dry cough, dyspnea, fatigue, nail clubbing
**Imaging Findings**	Ground-glass opacities, occasional fibrosis	Ground-glass opacities, centrilobular nodules, mosaic attenuation	Fibrosis, honeycombing, enlarged mediastinal lymph nodes
**Histopathology**	Inflammation and fibrosis (chronic cases)	Inflammation, fibrosis, granulomas	Fibrosis with usual interstitial pneumonia pattern
**Diagnostic Tools**	Clinical history, imaging response to drug cessation	Serum IgG for antigen exposure, biopsy showing granulomas	Clinical and radiologic findings, lung biopsy
**Prognosis**	Typically reversible upon drug cessation	Variable; can improve if antigen exposure is removed	Progressive, poor prognosis
**Treatment Response**	Rapid improvement (acute cases within 24 h, chronic within 3 months)	Improvement if antigen exposure is removed, but can be chronic	Poor response to treatment, progressive decline

**Table 3:** Strategies to minimize nitrofurantoin-associated pulmonary toxicity and optimize UTI management.

Strategy	Description
**Risk Assessment**	Identify high-risk populations, including elderly patients and those with renal impairment.
**Renal Function Monitoring**	Assess eGFR before prescribing; avoid use in patients with eGFR < 30 mL/min to prevent drug accumulation and toxicity.
**Baseline and Periodic Monitoring**	Evaluate renal, hepatic, and pulmonary function regularly, especially in long-term users, to detect early signs of toxicity.
**Alternative Treatments for Recurrent UTIs**	Consider alternative antibiotics (e.g., trimethoprim-sulfamethoxazole, fosfomycin, or cephalexin) based on resistance patterns and patient needs.
**Non-Antibiotic Preventive Measures**	Utilize probiotics, increased hydration, dietary modifications, cranberry supplements, and D-mannose to reduce UTI recurrence.
**Education and Awareness**	Train healthcare providers on risk factors, prescribing guidelines, and monitoring protocols to ensure safe use.
**Shared Decision-Making**	Involve patients in treatment decisions, discussing risks, benefits, and alternative options to promote informed choices.

## Data Availability

Data sharing is not applicable to this article as no datasets were generated or analyzed during the current study.
